# Pre- and postnatal brain magnetic resonance imaging in congenital cytomegalovirus infection: a case report and a review of the literature

**DOI:** 10.1186/s12887-022-03334-x

**Published:** 2022-05-18

**Authors:** Laurien Vanbuggenhout, Michael Aertsen, Luc De Catte, Gunnar Naulaers

**Affiliations:** 1 Department of Pediatrics, University Hospitals Leuven, Herestraat 49, 3000 Leuven, Belgium; 2 Department of Radiology, University Hospitals Leuven, Leuven, Belgium; 3 Department of Feto-Maternal Medicine, University Hospitals Leuven, Leuven, Belgium; 4 Neonatal Intensive Care Unit, University Hospitals Leuven, Leuven, Belgium

**Keywords:** Congenital cytomegalovirus infection, Brain, Magnetic resonance imaging, Calcifications, Neurological outcome

## Abstract

**Background:**

Congenital cytomegalovirus infection (cCMV) is the most common known viral cause of neurodevelopmental delay in children. The risk of severe cerebral abnormalities and neurological sequelae is greatest when the infection occurs during the first trimester of pregnancy. Pre- and postnatal imaging can provide additional information and may help in the prediction of early neurological outcome.

**Case presentation:**

This report presents the case of a newborn with cCMV infection with diffuse parenchymal calcifications, white matter (WM) abnormalities and cerebellar hypoplasia on postnatal brain imaging after magnetic resonance imaging (MRI) and neurosonogram (NSG) at 30 weeks showing lenticulostriate vasculopathy, bilateral temporal cysts and normal gyration pattern according to the gestational age (GA). No calcifications were seen on prenatal imaging.

**Conclusion:**

cCMV infection can still evolve into severe brain damage after 30 weeks of GA. For this reason, a two-weekly follow-up by fetal NSG with a repeat in utero MRI (iuMRI) in the late third trimester is recommended in cases with signs of active infection.

## Established facts and novel insights

Established Facts

• Congenital CMV infection is the most common known viral cause of neurodevelopmental delay in children.

• CMV infection can persist over a relatively long period of time during pregnancy thereby causing a combination of multiple brain lesions on iuMRI and/or fetal US.

• Fetal US remains the key investigation method in the follow-up of cCMV infection during pregnancy.

• Pre- and postnatal cranial US and brain MRI provide additional information and are important for predicting early neurological outcome.

Novel Insights

 • Congenital CMV infection can still evolve into severe brain damage after 30 weeks of GA.

• A two-weekly follow-up by fetal NSG with a repeat iuMRI in the late third trimester is indicated in cases where active infection is documented.

## Background

Cytomegalovirus (CMV) infection is the most common congenital infection worldwide with an estimated incidence in industrialized countries of 0.6 to 0.7% of all live births and 10 to 15% of those being symptomatic at birth. It is the leading non-genetic cause of sensorineural hearing loss (SNHL) and the most common known viral cause of neurodevelopmental delay in children [1–3].

Congenital CMV (cCMV) infection is caused by transplacental transmission of the virus and can result from a primary maternal infection or a non-primary infection (reactivation or reinfection by a different viral strain). The greatest risk of in-utero transmission is associated with primary CMV infections. There is no difference in audiological and neurodevelopmental outcome between infants born to mothers with a primary infection and those born to mothers with a non-primary infection [4–6]. The clinical presentation of cCMV infection varies widely, from an asymptomatic infection to a potentially life-threatening disseminated disease. Most common neonatal presentations of symptomatic disease are petechiae, jaundice, hepatomegaly, splenomegaly, microcephaly and other neurologic signs [3]. 

Due to more frequent serology testing and better prenatal ultrasound screening, the prenatal diagnosis of cCMV infection increased. Serology screening is recommended before 15 weeks of GA because a maternal infection in the first trimester can lead to severe long-term sequelae [7]. Maternal primary infection is identified by seroconversion. When seroconversion cannot be demonstrated, screening is based on IgG and IgM testing followed by IgG avidity testing in cases with positive IgM. A low IgG avidity index is suggestive of a recent primary infection [7–10]. In seropositive women, serological testing is not informative to confirm non-primary infection [8, 9]. The gold standard for prenatal diagnosis is a PCR for CMV DNA in the amniotic fluid because infected fetuses pass the virus in their urine. Timing of amniocentesis is crucial since the sensitivity for CMV detection is higher after 20 weeks of gestation and at least 6 to 8 weeks after the diagnosis of maternal infection with a sensitivity of 90 to 100% [7–10]. When cCMV infection is confirmed, fetal US is recommended every 2 to 4 weeks. Additionally, iuMRI can be done and will help to determine the prognosis [8, 9]. Severe cerebral lesions are correlated with a worse neurodevelopmental outcome [8].

In this article, we describe a case of cCMV infection where cerebral lesions evolved into severe brain damage after 30 weeks of GA.

## Case presentation

A 29-year-old Caucasian woman, gravida 3, para 1, abortion 1, gave birth to a female infant at 39 weeks and 1 day of GA. She is a kindergarten teacher with no previous medical history. She was only immune for rubella and toxoplasmosis before pregnancy. Other infectious serology (CMV, hepatitis B/C, HIV and syphilis) was negative. Pregnancy was complicated with CMV seroconversion during the first trimester. Amniocentesis at 20 weeks revealed a positive PCR for CMV DNA. NSG at GA of 22 weeks was negative. At 24 weeks, it showed a hyperechogenic periventrical zone bilaterally. This was confirmed at 26 and 27 weeks of gestation. In addition, also mild lenticulostriate vasculopathy was seen. iuMRI at 27 weeks of GA showed normal to slightly delayed gyration for the GA, bilateral small temporal cysts, a region of increased signal intensity at the crossroads against the basal ganglia and a discrete band-shaped zone of increased signal intensity cranial to the lateral ventricles, slightly more on the left side than on the right but with normal apparent diffusion coefficient (ADC) values on diffusion weighted imaging (DWI). NSG at 30 weeks showed more severe lenticulostriate vasculopathy and bilateral temporal cysts. The lateral ventricular lining was slightly irregularly shaped. A follow up iuMRI at 30 weeks was performed and confirmed the NSG findings with an improvement in gyration, now in accordance with the GA of 30 weeks. Parents were counseled about the risk of hearing loss and mild to moderate motor impairment. Severe to very severe motor dysfunction like cerebral palsy, was not expected. The follow up of the remainder of the pregnancy occurred in the referral hospital.

Labor was induced at 39 weeks and 1 day because of fetal growth arrest. Due to fetal distress and meconium stained amniotic fluid, a vacuum extraction was performed. The girl was well at birth, with an Apgar score of 9/9/10, and did not need immediate life support. She had a birth weight of 2440 g (percentile 3), a birth length of 44.5 cm (<P3) and a head circumference of 32 cm (P3-P10). Shortly after birth, she developed petechiae due to severe thrombocytopenia of 13 × 10^9^/L and hypoglycemia, for which she was transferred to a tertiary NICU center. 

**Fig. 1:**
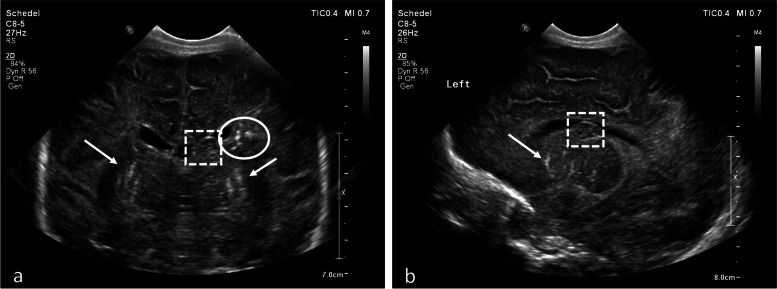
Postnatal cranial ultrasound, convex transducer. **a** coronal plane at the foramen of Monro. **b** parasagittal plane over the left ventricle. At day 1 of life lenticulostriate vasculopathy (white arrow), germinolysis (white square) and extensive periventricular calcifications (white circle) were visible

During hospitalization, she received a platelet transfusion on day 1, day 2, day 4 and day 7 of life. Hypoglycemia recovered with an adequate intake, initially parenteral then fully enteral, with a normal glucose load. cCMV infection was confirmed by a strongly positive urine PCR (> 5,000,000 CMV IU/mL). Brainstem Evoked Response Audiometry (BERA) was abnormal with suspicion of bilateral severe sensorineural hearing loss. There were no signs of CMV infection at funduscopic examination. Postnatal US of the brain at day 1 showed germinolysis, temporal cysts and extensive bilateral calcifications, mainly periventricular, previously not visualized on prenatal imaging (shown in Fig. 1). Oral valganciclovir was started on day 5.

**Fig. 2:**
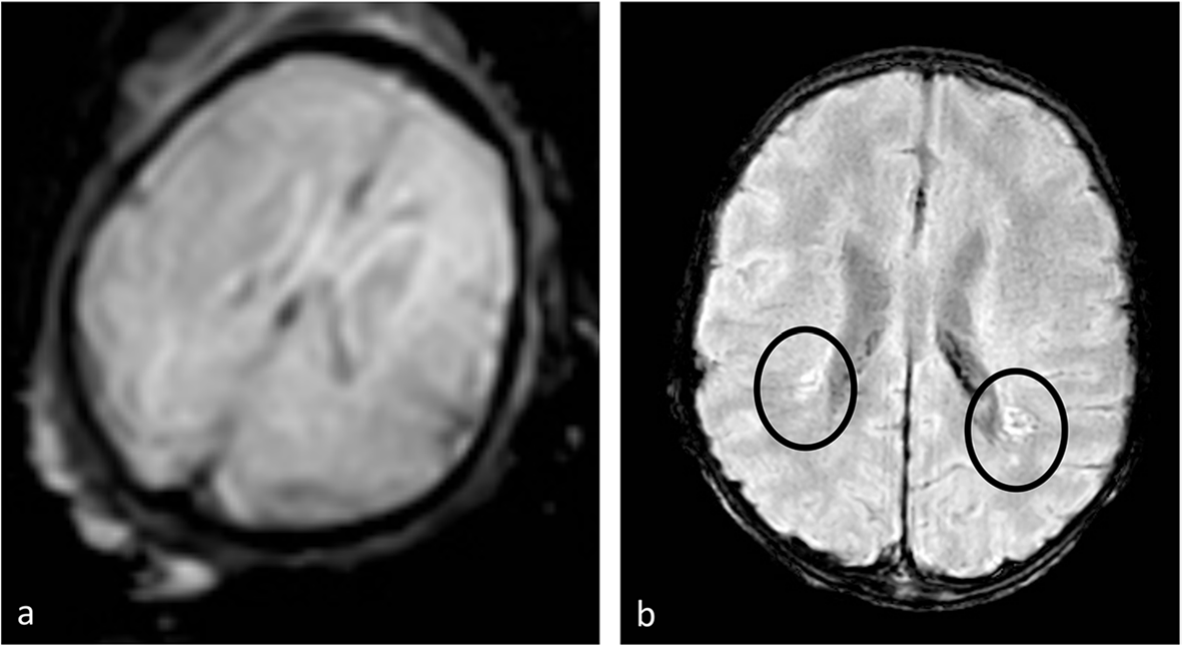
Magnetic resonance imaging. **a** Prenatal T2-weighted echo planar imaging (EPI) in the axial plane **b** Postnatal susceptibility weighted imaging (SWI) in the axial plane. Postnatally, the bilateral periventricular calcifications (black circle) are seen, not evident on in utero EPI images

**Fig. 3:**
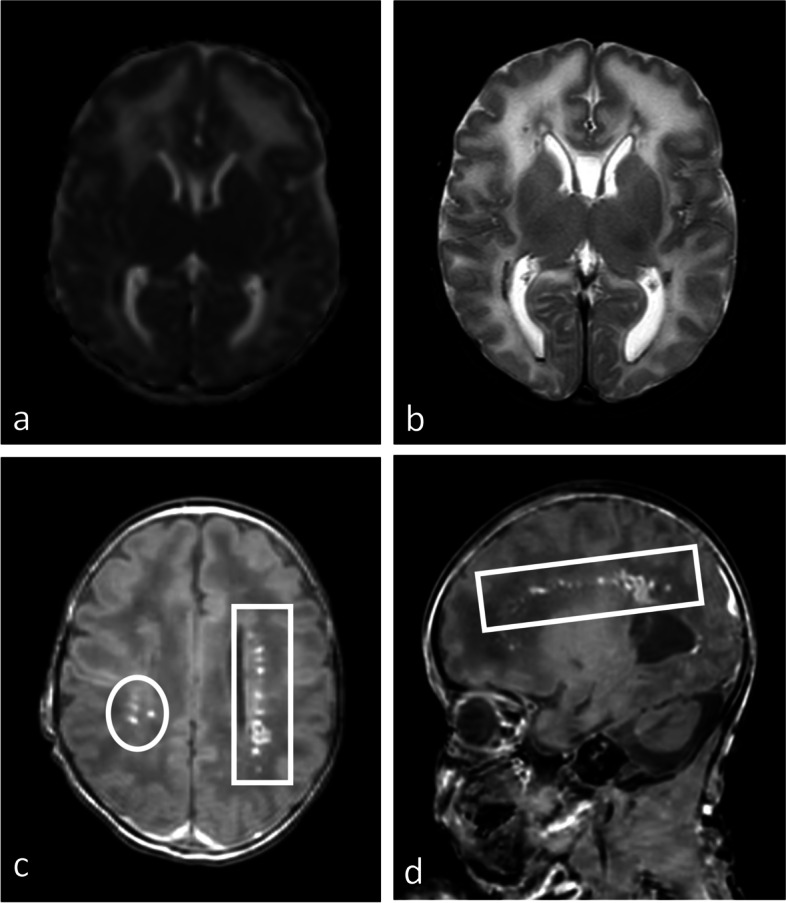
Postnatal MRI of the brain at day 19. **a** Apparent diffusion coefficient (ADC) map in the axial plane. **b** T2-weighted axial image. **c** T1-weighted axial plane. **d** T1-weighted sagittal plane. **a**, **b** Diffuse white matter signal alterations on T2 weighted images with increased ADC values on diffusion weighted imaging. **c**, **d** Extensive bilateral periventricular calcifications in the axial and sagittal plane (circle and square)

After discharge, the patient underwent a MRI of the brain on day 19 of life. MRI confirmed multiple periventricular and subependymal calcifications and showed a delayed myelination at the posterior limb of the internal capsule (PLIC), cerebellar hypoplasia, diffuse WM signal alterations with increased ADC on DWI and suspicion of polymicrogyria (shown in Figs. 2 and 3). All findings were compatible with cCMV infection. Furthermore, sequelae of intracranial haemorrhage, presumably related to the known cCMV-induced thrombocytopenia and the vacuum extraction, were seen on brain imaging.

Currently, the infant is 8 months old and receives physiotherapy twice a week. She has a mild motor development delay with limited hypertonia in the upper and lower limbs. There has been a recovery of the bilateral hearing loss with almost a normalization on the left side and persistent moderate SNHL on the right side. Therapy with oral valganciclovir was given for a total of 6 months.

## Discussion and conclusion

Congenital CMV infection weighs on the global healthcare system because of the significant risk of long-term sequelae, most commonly SNHL and neurodevelopmental delay. In the literature, evidence to support treatment decisions is still rare. Early predictors of poor outcome may determine the prognosis and management, and serve as aid in counseling parents. Several studies have been conducted to examine abnormal neuroradiological findings associated with cCMV infection and their prognostic role.

Neuroradiological findings found with cCMV are related to the pathogenesis of the disease. CMV reaches the brain by the blood and the cerebrospinal fluid causing meningitis and choroid plexitis in patients with mild cCMV infection. In severe cases, CMV particles diffuse into the parenchym and reach the ventricular and subventricular zones. These zones are characterized with dense neural stem progenitor cell [NSPC) populations. CMV inhibits NSPC proliferation and differentiation into neuronal and glial cells, resulting in malformation of cortical development and neuronal cell loss. In addition, neurotoxic factors produced by the infected glia cells cause focal parenchymal areas of necrosis and calcifications. Placental damage due to CMV infection causes placental insufficiency and hypoperfusion and may lead to fetal hypoxia which could contribute to the pathogenesis of some brain abnormalities such as polymicrogyria and WM lesions [7, 11, 12].

**Fig. 4:**
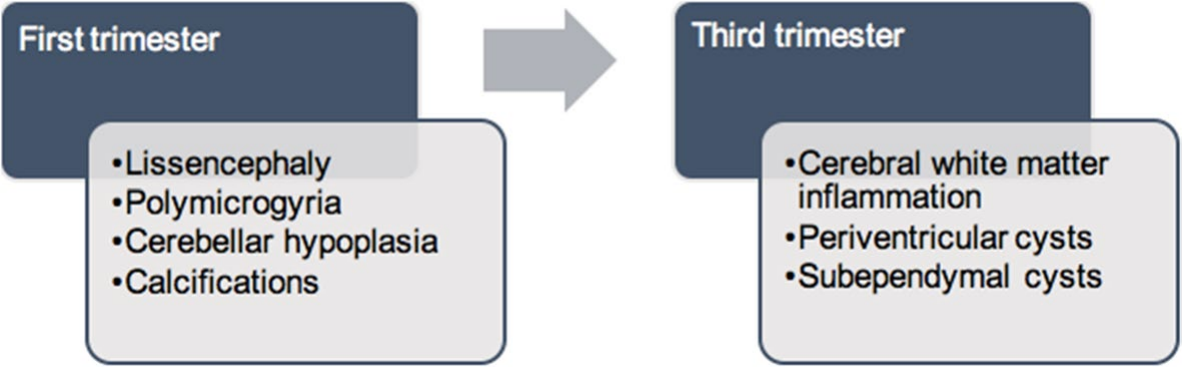
Neuroradiological findings according to time of infection

 In the fetus, neuronal proliferation starts between 8 and 16 weeks of gestation. The migration of these neurons to the cortex commences between 12 and 20 weeks, followed by cortical organization at 22 weeks of gestation. Near the end of neuronal production, generation of astrocytes begin. At 26 weeks GA astrocytes are the predominant cell type. In the first half of the third trimester astrocytes differentiate into oligodendrocytes. At 32 to 34 weeks the germinal zone becomes depleted. This explains why an early infection results in a reduction of both neurons and glia cells, whereas an infection after 22 to 24 weeks of gestation only affects the number of glia cells. An injury after 24 to 26 weeks does not affect the migration of neurons to the cerebral cortex because migration is already completed. In addition, it also clarifies why different neuroradiological findings can be found according to the time of infection during pregnancy. First trimester infections can cause lissencephaly, polymicrogyria, cerebellar hypoplasia and calcifications. Infections during third trimester, when neuronal migration and organization is finished, may lead to cerebral WM inflammation and periventricular/subependymal cysts (shown in Fig. 4). However, infection may persist over a relatively long time and may cause a combination of malformations and lesions [7, 13] .

Isolated intraventricular haemorrhage and lenticulostriate vasculopathy are other radiological findings that are often reported with cCMV infection. The first is a result of a CMV related thrombocytopenia or central nervous system vasculitis. The latter is a non-specific finding that can also be associated with genetic abnormalities, congenital toxoplasmosis, twin-to-twin transfusion syndrome and human immunodeficiency virus (HIV) [7].

Several studies have compared the diagnostic and prognostic role of iuMRI with prenatal US (transabdominal and/or transvaginal US). Doneda et al. [14] investigated the diagnostic capability of iuMRI and prenatal US in a cohort of 38 fetuses at a mean GA of 25 weeks. They showed that iuMRI has a greater sensitivity in predicting symptomatic infection and in detecting certain brain anomalies than prenatal US at early GA. Especially polar temporal lesions, microencephaly and cortical malformations are better detected on iuMRI early in pregnancy. They demonstrated that a repeat iuMRI in the mid third trimester can be valuable for searching new pathological findings and for monitoring the evolution of previously diagnosed lesions. Finally, the study revealed low positive predictive values (PPV) in predicting CMV infection-related postnatal symptoms for both imaging techniques but high negative predictive values (NPV). On the other hand, Benoist et al. [15] showed that fetal MRI in combination with fetal US has a higher PPV for detecting CMV-related fetal brain abnormalities than separately. They proposed to use fetal MRI complementary to US. A prospective study of Lipitz et al. [16] showed that normal brain findings on fetal US and iuMRI are associated with a good neonatal outcome but do not rule out the risk of SNHL.

In conclusion, fetal US remains the key investigation method in the follow-up of cCMV infection but its utility is limited by the late manifestation of cranial and extracranial abnormalities [17]. US abnormalities can appear 12 weeks or more after a maternal infection. Therefore, a fetal US is recommended every 2 to 4 weeks in fetuses with confirmed cCMV infection [8]. iuMRI should be considered as a complementary imaging modality for investigating the fetal brain and improves the positive and negative predictive value of fetal US [8, 17]. Up to now, no studies have been published on the frequency of brain lesions occurring in the late third trimester on NSG and/or iuMRI in women with a first trimester cCMV infection.

Postnatal cranial US is a useful, widely accessible, low cost and harmless imaging technique for detecting brain abnormalities in infants with cCMV infection. However, abnormalities in the cortex, WM and posterior cranial fossa are difficult to detect with US techniques and the interpretation depends on the skills and the expertise of the radiologist. Postnatal brain MRI has replaced the head computed tomography (CT) because it is better in detecting WM abnormalities and migration disorders without exposure to radiation and it improves prediction of neurological outcome. The evaluation of WM abnormalities on MRI is challenging, especially if myelination is not yet completed. The prognosis of isolated mild WM disease has not yet been well determined [18–20].

Several studies have investigated the correlation between early postnatal brain MRI abnormalities and neurodevelopmental outcome (shown in Table 1). The results of these studies should be interpreted with care because of the small sample size, the retrospective design or the inclusion of infants with symptomatic cCMV infection only. The studies also vary in their methods of diagnosing fetal infection, imaging surveillance regime, postnatal follow-up periods and reported outcomes. Many studies do not document about the timing of maternal infection and the prevalence of terminations of pregnancy. Capretti et al. [18] assessed the role of cranial US and MRI in 40 neonates with cCMV infection. Neonates with a normal postnatal brain MRI had a high likelihood for having a normal auditory- and neurodevelopment. By contrast, an abnormal brain MRI, even with normal US findings, suggested a high likelihood of SNHL or developmental delay. A retrospective study by Manara et al. [19] of MRI findings in 14 children with symptomatic cCMV infection identified cortical malformations, ventriculomegaly and hippocampal dysplasia as strong predictors for severe neurologic impairment. Kwak et al. [21] predicted neurodevelopmental outcome based on MRI findings only and showed that ventriculomegaly, WM abnormalities and calcifications are associated with epilepsy. Polymicrogyria, a migration disorder, is related with epilepsy and developmental delay. SNHL is not associated with any abnormal finding on brain MRI. Nishida et al. [22] showed that infants with at least 2 of ventriculomegaly, periventricular cysts and WM injury may be at high risk of developing neurodevelopmental impairment. Other studies developed a scoring system to predict neurological outcome. The study by Alarcon et al. [23] proposed a new scoring system based on Noyola’s CT scoring systems. They combined brain US and MRI findings to predict neurodevelopmental outcome in 16 children with symptomatic cCMV. A score of ≥ 2 according to the new Noyola’s scale was associated with a significant risk of death or moderate/severe disability. Lucignani et al. [24] constructed a new MRI severity score to predict long-term neurological outcome in children with symptomatic and asymptomatic cCMV infection. The presence of WM alterations and ventriculomegaly is associated with an increased risk of adverse neurological outcome, especially if the MRI score is > 2. In a recently published study of Vande Walle et al. [25] neonatal brain MRI showed abnormalities in more than 30% of clinically asymptomatic and 75% of symptomatic newborns with cCMV. Thereby proving the importance of performing brain MRI in all newborns with cCMV, even if they are asymptomatic. The study is one of the largest cohorts investigating MRI abnormalities in both symptomatic and asymptomatic newborns with cCMV. WM lesions are the most common abnormality observed on brain MRI in the study, followed by subependymal cysts and ventriculomegaly. Migration abnormalities, calcifications and cerebellar anomalies were seen in 15–25% of clinically symptomatic patients but were very rare in asymptomatic patients. Most patients showed multiple brain lesions associated with cCMV which supports the theory that prenatal infection can persist for some time resulting in a combination of lesions. Unfortunately, neurological outcome was not investigated in this study.

**Table 1:** Correlation between postnatal brain abnormalities on imaging and neurodevelopmental outcome

Study	Study design	Study population	Type of imaging	Predictors of neurological outcome
**Capretti et al.** [18]	Prospective longitudinal observational study	40 (symptomatic and asymptomatic)	Cranial US and brain MRI	Abnormal brain MRI
**Manara et al.** [19]	Retrospective study	14 (symptomatic)	Brain MRI	Cortical malformations, ventriculomegaly or hippocampal dysplasia
**Kwak et al.** [21]	Retrospective study	31 (symptomatic)	Brain MRI	Polymicrogyria, ventriculomegaly, WM abnormalities or calcifications
**Nishida et al.** [22]	Prospective cohort study	42 (symptomatic and asymptomatic)	Brain MRI	≥ 2 of ventriculomegaly, periventricular cysts or WM injury
**Alarcon et al.** [23]	Prospective longitudinal observational study	26 (symptomatic)	Cranial US, CT and brain MRI	≥ 2 according to the new Noyola’s scale
**Lucignani et al.** [24]	Retrospective study	44 (symptomatic and asymptomatic)	Brain MRI	MRI score > 2, especially WM alterations and ventriculomegaly

In the future, long-term prospective multicenter follow-up cohort studies with a large sample size including both symptomatic and asymptomatic neonates with cCMV infection, are necessary to correlate MRI findings with neurodevelopmental outcome without inclusion of SNHL.

Congenital CMV infection is the most common known viral cause of neurodevelopmental delay in children and has a large impact on the global healthcare system. Currently, evaluation of the prognosis of cCMV infection is based mainly on the presence of fetal and postnatal brain lesions on imaging (US and MRI). When fetal US shows signs of cCMV infection, iuMRI is recommended during pregnancy. A repeat MRI in the mid third trimester could be valuable to search for new pathologic findings, to monitor the evolution of previously diagnosed lesions and to help with early neurological prognosis. According to previous studies cortical malformations, ventriculomegaly, WM abnormalities and calcifications are associated with a worse neurological outcome.

In conclusion, this case report shows that a primary CMV infection in the first trimester of pregnancy is able to deteriorate after 30 weeks of GA calling for a two-weekly follow-up by fetal NSG with repeated iuMRI in the late third trimester to enhance prognostication of neurodevelopmental outcome.

## Data Availability

All data analyzed during this study are included in this article. Further enquiries can be directed to the corresponding author.

## Abbreviations

cCMV: Congenital cytomegalovirus
WM: White matter
MRI: Magnetic resonance imaging
NSG: Neurosonogram
GA: Gestational age
iuMRI: In utero MRI
CMV: Cytomegalovirus
SNHL: Sensorineural hearing loss
US: Ultrasound
ADC: Apparent diffusion coefficient
DWI: Diffusion weighted imaging
P: Percentile
BERA: Brainstem Evoked Response Audiometry
PLIC: Posterior limb of the internal capsule
NSPC: Neural stem progenitor cell
HIV: Human immunodeficiency virus
PPV: Positive predictive values
NPV: Negative predictive values
CT: Computed tomography
